# Molecular diagnostic approaches for enteric parasites: an issue of relevance for deployed soldiers?

**DOI:** 10.1186/s40779-022-00391-2

**Published:** 2022-06-09

**Authors:** Hagen Frickmann

**Affiliations:** 1 Department of Microbiology and Hospital Hygiene, Bundeswehr Hospital Hamburg, 22529 Hamburg, Germany; 2 Institute for Microbiology, Virology and Hygiene, University Medicine Rostock, 18057 Rostock, Germany

**Keywords:** Polymerase chain reaction (PCR), Test evaluation, Infection, Deployment, Military medicine, Surveillance, Screening, Diagnostic accuracy

Dear Editor,

Recently, Lindrose et al. [1] have conducted a retrospective assessment over 6 years on enteric helminth infections in United States (US) soldiers and their relatives to provide epidemiological insights into this so far neglected topic. A total of 50,000 helminth infections were recorded during the observation period. Next to classically hygiene-related infections like enterobiasis, infections due to soil-transmitted nematodes like *Strongyloides stercoralis*, waterborne trematode infections like schistosomiasis as well as invasive cestode infections like neurocysticercosis were among the more frequently recorded diagnoses. Of note, systematic screening was not conducted in the assessed US soldiers after deployments to tropical areas of endemicity. Insofar, it can only be speculated how many asymptomatic infections went undetected and, as concluded by the authors, whether systematic returnee screenings seem justified or not.

The reliability of the results of such screening approaches, however, necessarily depends on the correctness of the obtained diagnostic results. Traditional parasitological diagnosis is based on microscopical assessments with previous enrichment steps, which are performed to increase the likelihood of seeing morphologically unambiguous structures of the parasites. However, microscopy remains a highly investigator-dependent diagnostic approach and the risk of overlooking parasites especially in complex sample material like stool is reciprocally correlated with the investigator’s experience. Particularly for protozoan parasites in stool, concordance of microscopic diagnostic results even between experienced European reference centers left considerable room for improvement in a recent assessment by Utzinger and colleagues [2]. The authors suggested that continued external quality assessments and the establishment of networks might improve diagnostic accuracy. If sufficient training cannot be maintained, e.g., due to decreasing availability of positive sample materials, a shift to less investigator-depending diagnostic assays may be considered.

Real-time polymerase chain reaction (PCR) is such a diagnostic technique, which combines sensitivity with inter-laboratory reproducibility. In Europe, particularly Dutch scientists early started with the assessment of real-time PCR for the diagnosis of parasitic infections. Consequently, the Dutch were the first to introduce an international external quality control assessment scheme for nucleic acid amplification testing for common enteric helminths in human stool samples as published by Cools et al. [3] in 2020. Disappointingly, however, the authors concluded that similarly as observed earlier for microscopy [2], considerable discrepancy of the results achieved with the different nucleic acid extraction and amplification assays was recorded, calling for further improvement. While real-time PCR for enteric protozoa is considered as well-established and provides additional diagnostic value, e.g., by discriminating cysts of *Entamoeba histolytica* and *Entamoeba dispar*, optimization of molecular diagnostic assays for helminths is still ongoing. It comprises not only the nucleic acid amplification assays themselves but also homogenization steps with the sample materials in order not to miss non-homogeneously distributed pathogen DNA within the samples as well as the nucleic acid extraction approaches to release target DNA from hard egg shells and cuticula cells prior to nucleic acid amplification. This ongoing need for further optimization is reflected by the yet improvable results of the Dutch external quality control assessment scheme [3].

Of note, the helminth nucleic acid amplification assays applied for the Dutch external quality control assessment scheme were primarily investigator-developed, so assay-specific weaknesses are likely to have relevantly contributed to the observed discrepancy [3]. To circumvent the problem of severe discrepancy of diagnostic accuracy of the assays in use, the European Union (EU) enforced the Regulation EU 2017/746 [Conformité Européenne-in vitro diagnostics (CE-IVD) regulation] in order to drastically increase the quality of the diagnostic process, which implies laboratories carrying out investigator-developed (in-house) assays with human sample materials should be accredited.

Funded by the German Ministry of Defense (grant number 36K2-S-45 1922), the Bundeswehr medical service participated in multicentric evaluation studies in the laboratories of the ISO 15189—accredited institutions Bernhard Nocht Institute for Tropical Medicine Hamburg and Institute for Medical Microbiology, Virology and Hygiene, University Medicine Rostock, with the aim of assessing the performance characteristics of commercial and in-house real-time PCR assays targeting enteric protozoa and parasites in several hundreds of samples with high pretest probability for parasitic infections with and without parallel microscopic investigations [4, 5]. Although all PCR assays were run with the same nucleic acid eluates to ensure identical reaction conditions during the assessments, concordance of the different assays was imperfect and varied considerably in a species-depending manner. Thereby, it could be confirmed that real-time PCR was more sensitive than traditional microscopy as suggested in the study by Utzinger et al. [2] and that the overall performance characteristics of the compared molecular assays were quite similar [4, 5], generally justifying their use. However, imperfect concordance of positive results with partly considerable 95% confidence intervals for the estimations of traditional test performance characteristics like sensitivity and specificity [4, 5] still demands expertise in the interpretation of respective diagnostic results.

Due to residual uncertainties on the diagnostic accuracy of the molecular diagnosis of enteric parasites as described by Cools et al. [3] and confirmed by recent evaluations [2, 4, 5], the question initially raised by Lindrose et al. [1] on the appropriateness of diagnostic screenings for enteric parasites in stool samples of soldiers after deployment in areas of endemicity remains unanswered. A combination of low pretest probability [1] and imperfect diagnostic accuracy [2–5] bears the risk of poor predictive values according to Bayes’ theorem in case of testing without preselection guided by clinical suspicion. Accordingly, ongoing optimization of molecular testing for enteric parasites, ideally in multicentric settings, will remain an issue in order to more thoroughly define the impact of respective infections on the health of deployed soldiers.

## Data Availability

Not applicable.

## Abbreviations

CE-IVD: Conformité Européenne-in vitro diagnostics
EU: European Union
PCR: Polymerase chain reaction
US: United States
